# Nomogram based on TNM stage to predict the prognosis of thymic epithelial tumors (TETs) patients undergoing extended thymectomy

**DOI:** 10.3389/fsurg.2023.1136166

**Published:** 2023-03-03

**Authors:** Yanzhi Li, Zhanpeng Tang, Xirui Zhu, Hui Tian

**Affiliations:** Department of Thoracic Surgery, Qilu Hospital of Shandong University, Jinan, China

**Keywords:** thymic epithelial tumors (TETs), nomogram, prognostic factor, TNM stage, cancerspecific survival (CSS)

## Abstract

**Background:**

Thymomas and thymic carcinoma are thymic epithelial tumors (TETs) of the anterior mediastinum. On the basis of The AJCC 8th Edition of TNM classification, no prognostic prediction model has been established for TETs patients undergoing surgical resection. In this study, based on data from Qilu Hospital of Shandong University, we identified prognostic factors and developed a nomogram to predict the prognosis for TETs patients undergoing extended thymectomy.

**Methods:**

Patients with TETs who underwent thymectomy between 2010 and 2020 were consecutively enrolled. An analysis of multivariate Cox regression and stepwise regression using the Akaike information criterion (AIC) was conducted to identify prognostic factors, and a nomogram for TETs was derived from the results of these analyses. The model was validated internally with the Kaplan-Meier curves, ROC curves and calibration curves.

**Results:**

There were 350 patients with TETs enrolled in the study, and they were divided into a training group (245,0.7) and a validation group (105,0.3). Age, histological type, tumor size, myasthenia gravis, and TNM stage were independent prognostic factors for CSS. The Kaplan-Meier curves showed a significant difference between high nomorisk group and low nomorisk group. A nomogram for CSS was formulated based on the independent prognostic factors and exhibited good discriminative ability as a means of predicting cause-specific mortality, as evidenced by the area under the ROC curves (AUCs) of 3-year, 5-year, and 10-year being 0.946, 0.949, and 0.937, respectively. The calibration curves further revealed excellent consistency between the predicted and actual mortality when using this nomogram.

**Conclusion:**

There are several prognostic factors for TETs. Based on TNM stage and other prognostic factors, the nomogram accurately predicted the 3-, 5-, and 10-year mortality rates of patients with TETs in this study. The nomogram could be used to stratify risk and optimize therapy for individual patients.

## Introduction

Thymomas and thymic carcinoma are both thymic epithelial tumors (TETs), which are relatively rare anterior mediastinal tumors. The WHO classifies TETs into five types: A, AB, B1, B2, B3, and TC. Type A/AB/B1 is a low-risk group with excellent overall survival (OS), and the 10-year overall survival rate is over 90%-95%. B2/B3/TC is a high-risk group, with 5-year survival rates of 75%, 70%, and 48%, respectively (1).

At present, the Masaoka-Koga staging system and American Joint Commission on Cancer (AJCC) 8th Edition of TNM classification are the two most commonly used staging systems for TETs. The Masaoka-Koga staging system relies primarily on primary tumor extension and the degree of involvement beyond the thymus (2). The AJCC 8th edition of the TNM classification, based on the combination of primary tumor local invasion, nodal involvement and metastatic spread, has been confirmed to play an important role in the diagnosis and treatment of TETs (3).

Currently, surgical resection remains the optimal treatment for TETs. Complete resection is of prognostic importance for patients with thymoma at any stage (4, 5).The standard surgical approach for stage I or II thymic tumors is thymectomy, in which the entire thymus is removed along with the tumor. Currently, extended thymectomy has been used because thymic tissue is often present in the mediastinal fat and may contribute to the non-remission of postoperative myasthenia gravis or the development of postoperative myasthenia gravis (1). Most patients can achieve satisfactory outcomes after extended thymectomy. In clinical treatment, patients with advanced stages are often treated with radiotherapy and chemotherapy after surgery. Studies have shown that postoperative radiotherapy for Masaoka-Koga stage III/IV could improve OS (6).

The well-recognized prognostic factors for TETs include tumor stage and resection status (5, 7, 8), and studies have reported that age, completeness of resection, and histological type are also important prognostic factors except staging (7–9). At present, a few nomograms have been established to predict the prognosis of TETs. Zhang et al. (10) established a prediction model based on the SEER database, but there are shortcomings such as excessive missing data, which affects the integrity and accuracy of the predictive model. In this study, we aimed to establish an effective prognostic prediction model based on TNM stage and other important clinicopathological parameters for TETs patients following extended thymectomy and provide a reference for patient postoperative therapy.

## Materials and methods

### Patient selection

The study was approved by the Qilu Hospital of Shandong University institutional review board (KYLL-202008-023-1). Written informed consent was signed by all patients to obtain their clinical information.

**Figure 1:**
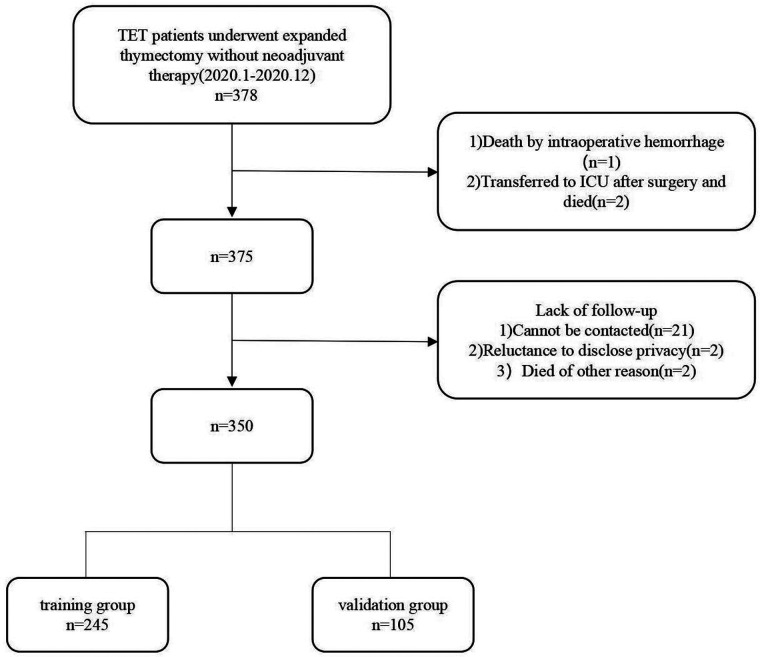
The flow diagram of the patients enrolled in this study. TET, Thymic epithelial tumor.

From January 2010 to December 2020, a total of 378 patients were diagnosed with TETs. In this study, 350 patients were treated with extended thymectomy, surgical approaches include median sternotomy and Video-Assisted Thoracic Surgery, recovered and were discharged (Figure 1).

The inclusion criteria are shown in Table 1.

**Table 1:** Inclusion criteria.

The inclusion criteria for this study
• Adult patients (age ≥18 years) who underwent extended thymectomy • Pathological diagnosis was TET • Detail medical records of patients could be allowed • Active follow-up with survival time and survival status, and definite cause of death

### Variables definition

The following data of eligible patients were collected from the database of Qilu Hospital: age at diagnosis, sex, histological type, tumor size, pleural effusion, lymph node dissection, positive lymph nodes, myasthenia gravis, surgical margin, TNM stage, postoperative radiotherapy, and postoperative chemotherapy. Some of the variables were regrouped, such as the age of diagnosis, which was divided into “<50” and “≥50”, since 50 years old was considered an important point(10, 11). Histological type was determined by the patient's pathology report and regrouped into “A/AB/B1”, “B2”, and “B3/CA”, because they were considered as low risk, intermediate risk and high risk groups for aggressiveness, recurrence and survival, respectively (1, 11–14). The cutoff point of tumor size (6.0 cm) was determined using X-tile (version 3.6.1) by Kaplan-Meier curve, in other studys, the cutoff point is selected as 5.5 or 6.6 cm (15, 16), which are close to our cutoff point, and tumor size was then divided into “<6 cm” and “≥6 cm”. For surgical resection margin, emphasis on completeness of excision (1), was divided into “R0” and “R1/R2”. The TNM stage was determined by the intraoperative findings and pathology reports each patient and was divided into “I”, “II”, and “III/IV”, as the invasion of adjacent organs in T satge(resectable or unresectable)(17, 18), is similar to the grouping of other studies with masaoka staging(15). The primary endpoint of the study was cancer-specific survival (CSS), which was measured from the time of diagnosis to (1) death from TETs and (2) the last follow-up.

### Statistical analysis

The multivariate Cox regression model and stepwise regression based on the Akaike information criterion (AIC) were used to explore the prognostic factors of TETs and select important variables for the model, and then shown as a forest map. Once the model was established, we used it to predict risk, and the effect of the prediction was assessed using the Kaplan-Meier curves, time-dependent receiver operating characteristic (tdROC) curves and calibration curves. The above analyses were performed using R, version 4.0.4 (R Foundation for Statistical Computing, Vienna, Austria) by package survminer, survival, rms, foreign, ggDCA, car, timeROC, ggforest and ggplot2. Variables were described using the medians [IQR] and numbers (%). Differences in these variables were assessed by the chi-squared or Fisher exact test. The analyses were performed using IBM SPSS Statistics 20. The hypothesis tests were two-sided, and *p* < 0.05 was considered to be statistically significant.

## Results

### Baseline characteristics

According to the inclusion criteria, 350 patients were enrolled in this study. The demographic, tumor and treatment characteristics of this cohort are shown in Table 2. There was no statistical difference between the inclusion and exclusion groups(Supplementary Table S1). Overall, the majority of the patients were ≥50 years old (210, 60%). In terms of treatment, most patients had surgical margins of R0 (329, 94%), no lymph node dissection (292, 83.4%), radiotherapy (117, 33.4%) or chemotherapy (55, 15.7%). In Table 2, from the perspective of survival status, among the surviving patients, most patients had the histological type A/AB/B1 (138, 42.9%) and a tumor size <6 cm (207, 64.3%). The surgical margin of most patients was R0 (312, 96.9%), Masaoka-Koga stage I/II (200, 62.1%), and TNM stage I (266, 82.6%). Among the deceased patients, most were histological type B3/CA (21, 75%), Masaoka-Koga stage III/IV (25, 89.3%), and TNM stage III/IV (15, 50%). In the majority of the patients who died, they died because of postoperative recurrence (24, 85.7%). The patients included in the study had a median follow-up of 45 months (interquartile range, 5–133 months). As of the last follow-up, 28 patients (8.0%) had died during the follow-up period, all from Ts and TC.

**Table 2:** Baseline characteristics of participants by Status.

Variable	Survival	Death	Total	*P* Value
N	322	28	350	
Age less than 50	133 (41.3)	7 (25.0)	140(40.0)	0.14
Male	156 (48.5)	21 (75.0)	177(50.6)	0.012
Histological				
A/AB/B1	138 (42.9)	3 (10.7)	141(40.3)	
B2	102 (31.7)	4 (14.3)	106(30.3)	
B3/CA	82 (25.5)	21 (75.0)	103 (29.4)	<0.001
Size less than 6	207 (64.3)	14 (50.0)	221(63.1)	0.19
Hydrothorax	73 (22.7)	4 (14.3)	77(22.0)	0.430
Lymph node dissection	51 (15.8)	7 (25.0)	58 (16.6)	0.32
Positive lymph node	3 (0.9)	2 (7.1)	5(1.4)	0.068
Myasthenia	117 (36.3)	11 (39.3)	128(36.6)	0.92
Margin R0	312 (96.9)	17 (60.7)	329 (94.0)	<0.001
Masaoka				
I/IIa	200 (62.1)	2 (7.1)	202(57.7)	
IIb	49 (15.2)	1 (3.6)	50(14.3)	
III/IV	73 (22.7)	25 (89.3)	98 (28.0)	<0.001
TNM				
I	266 (82.6)	3 (10.7)	269(76.9)	
II	44 (13.7)	11 (39.3)	55(15.7)	
III/IV	12 (3.7)	14 (50.0)	26 (7.4)	<0.001
Radiotherapy	100 (31.1)	17 (60.7)	117(33.4)	0.003
Chemotherapy	36 (11.2)	19 (67.9)	55(15.7)	<0.001
Relapse	9 (2.8)	24 (85.7)	33 (9.4)	<0.001
Follow-up duration	44.0 [23.0, 79.8]	48.0 [27.3, 79.8]		0.73

TNM, Tumor Node Metastasis;Continuous variables are presented as median [IQR]. Categorical variables are presented as *n* (%).

### Cox multivariate regression and stepwise regression

**Figure 2:**
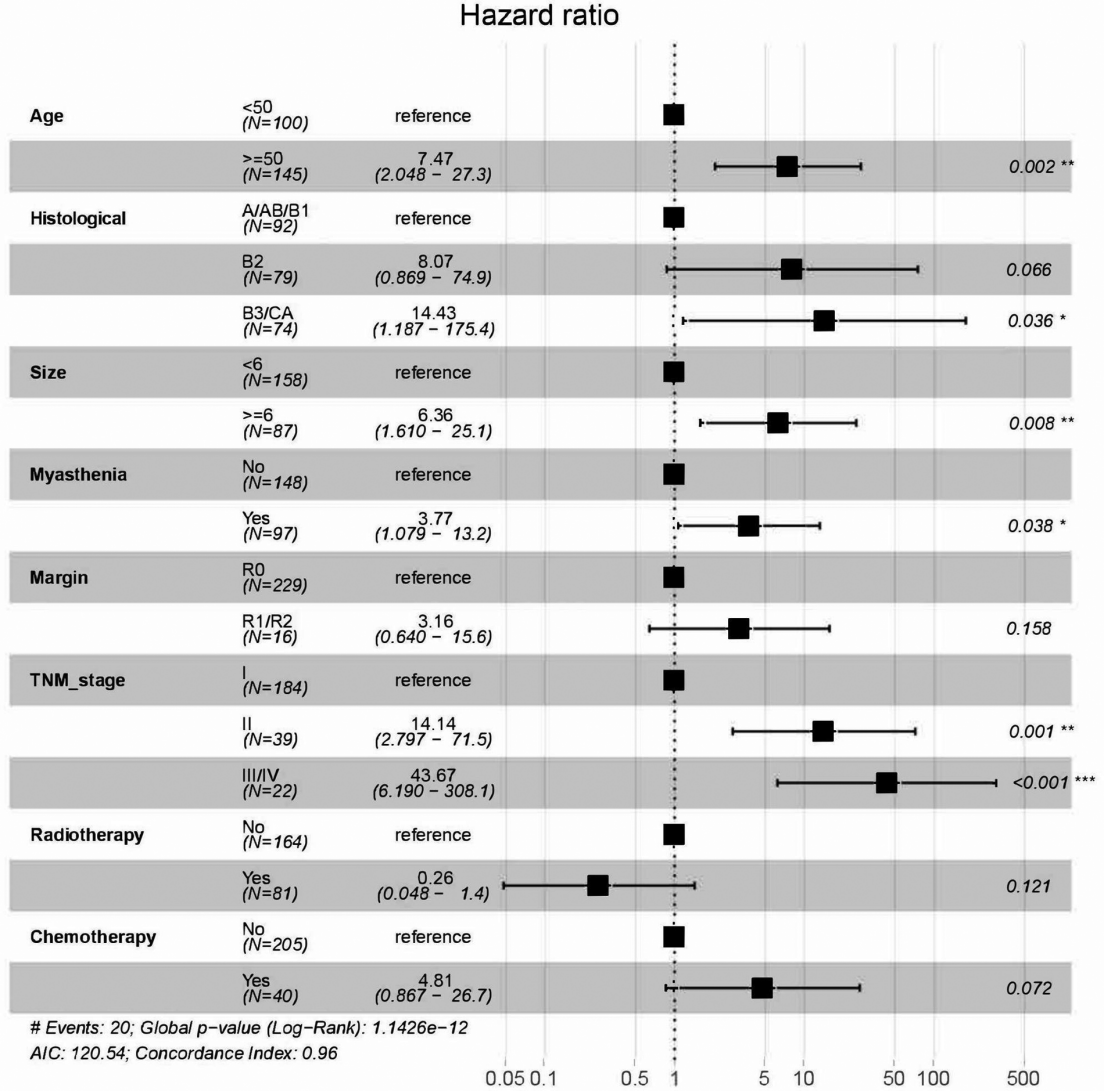
Hazard ration of variables based on multivariate cox regression model *and stepwise regression* based on the Akaike information criterion.

There were 350 patients with Ts or TC enrolled in the study, and the patients were divided into a training group (245,0.7) and a validation group (105,0.3). The multivariate Cox regression model was used to explore the prognostic risk factors for TET, and stepwise regression based on AIC was used to select important variables for the model. After screening, age, histological type, tumor size, myasthenia gravis, surgical margin, TNM stage, radiotherapy, and chemotherapy were important variables, except for surgical margin, radiotherapy, and chemotherapy, which were all statistically significant (Table 3). Age ≥ 50(HR = 7.47, *P* = 0.002), histological type B3/Ca(HR = 14.4, *P* = 0.036), tumor size ≥6 cm(HR = 6.36, *P* = 0.008), myasthenia gravis(HR = 3.77,*P* = 0.038), TNM stage II (HR = 14.1, *P* = 0.001) or III/IV (HR = 43.7, *P* < 0.001) were risk factors (Figure 2).

**Table 3:** Multivariate Cox regression model analyses of CSS in the nomogram cohort.

Variables	HR (95% CI)	Estimate	Std Error	*P* Value
Age more than 50	7.47 (2.05, 27.3)	2.01	0.6604	0.002
Histological				
A/AB/B1	Reference	-	-	
B2	8.07 (0.87, 74.9)	2.08	1.1370	0.066
B3/CA	14.4 (1.18, 175)	2.67	1.2744	0.036
Size more than 6	6.36 (1.61, 25.1)	1.85	0.7006	0.008
Myasthenia	3.77 (1.08, 13.2)	1.33	0.6379	0.038
Margin R1/R2	3.16 (1.08, 15.6)	1.15	0.8144	0.16
TNM stage				
I	Reference	-	-	
II	14.1 (2.80, 71.5)	2.65	0.8268	0.001
III/IV	43.7 (6.19, 308)	3.78	0.9968	<0.001
Radiotherapy	0.26 (0.05, 1.43)	−1.35	0.8673	0.12
Chemotherapy	4.81 (0.87, 26.7)	1.57	0.8739	0.072

TNM, Tumor Node Metastasis; HR, hazard ratio.

### Prognostic nomogram for CSS and validations

**Figure 3:**
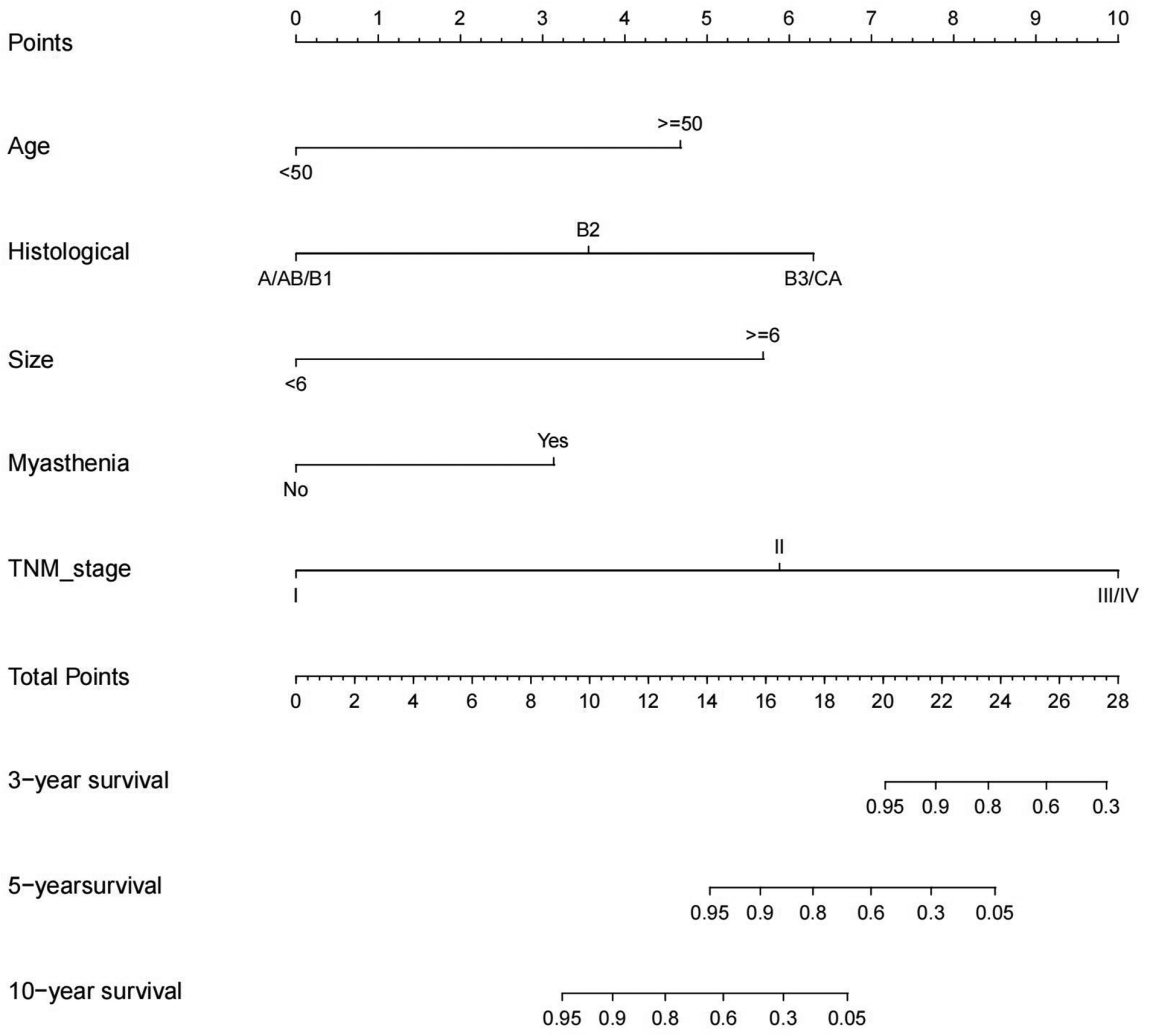
Competing risk nomogram for the prediction of 3-, 5-, 10-year cause-specific survival associated with TETs.

The significant variables in the multivariate Cox regression analysis and stepwise regression based on AIC were included in the nomogram, and each variable was given a score according to the HR (Figure 3). Then, by summing the total scores for each variable and locating them on a total subscale, the probability of CSS at 3 and 5 years and 10 years for the patients was derived. For example, if a 65-year man with type B2, TNM stage III, tumor size 4.5 cm, and myasthenia gravis underwent extended thymectomy, he would score 17 points, which means that this patient has an approximately 80% possibility of survival in the fifth year and an approximately 15% possibility of survival in the tenth year.

**Figure 4:**
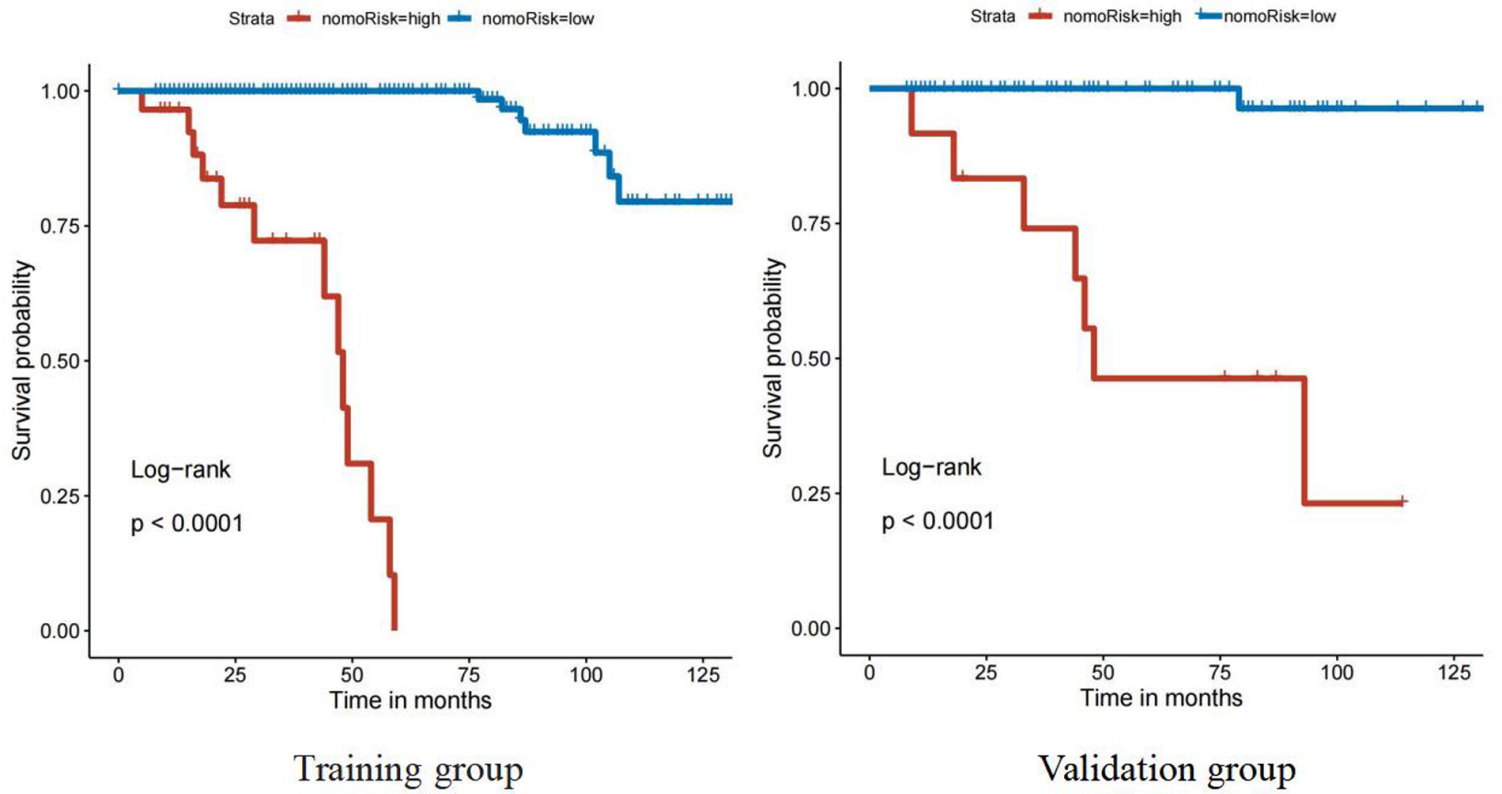
The Kaplan-Meier curves of training group and validation group.

**Figure 5:**
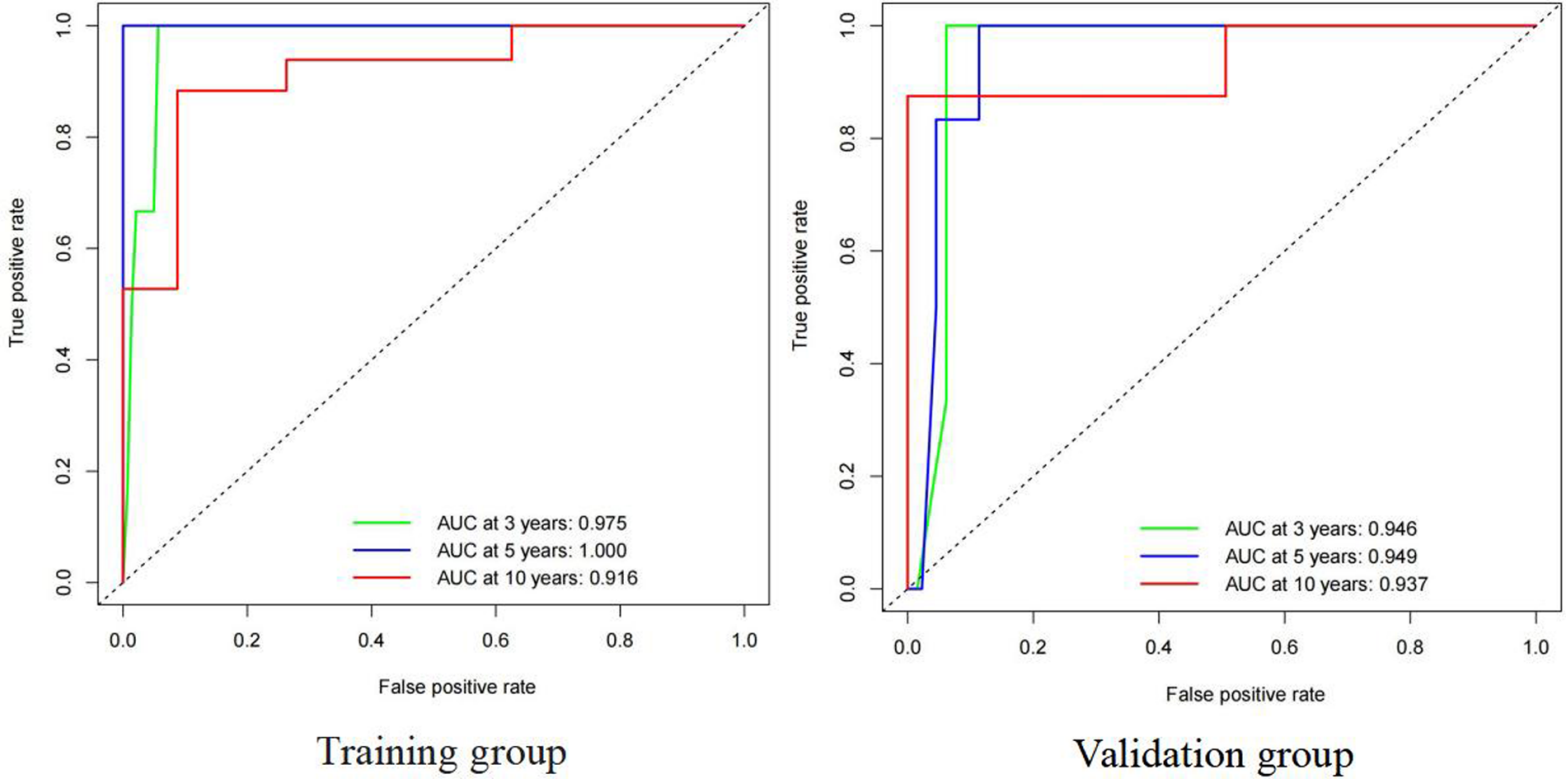
Time-dependent receiver operating characteristic (ROC) curve for cause-specific survival nomogram in TETs of training group ang validation group.

**Figure 6:**
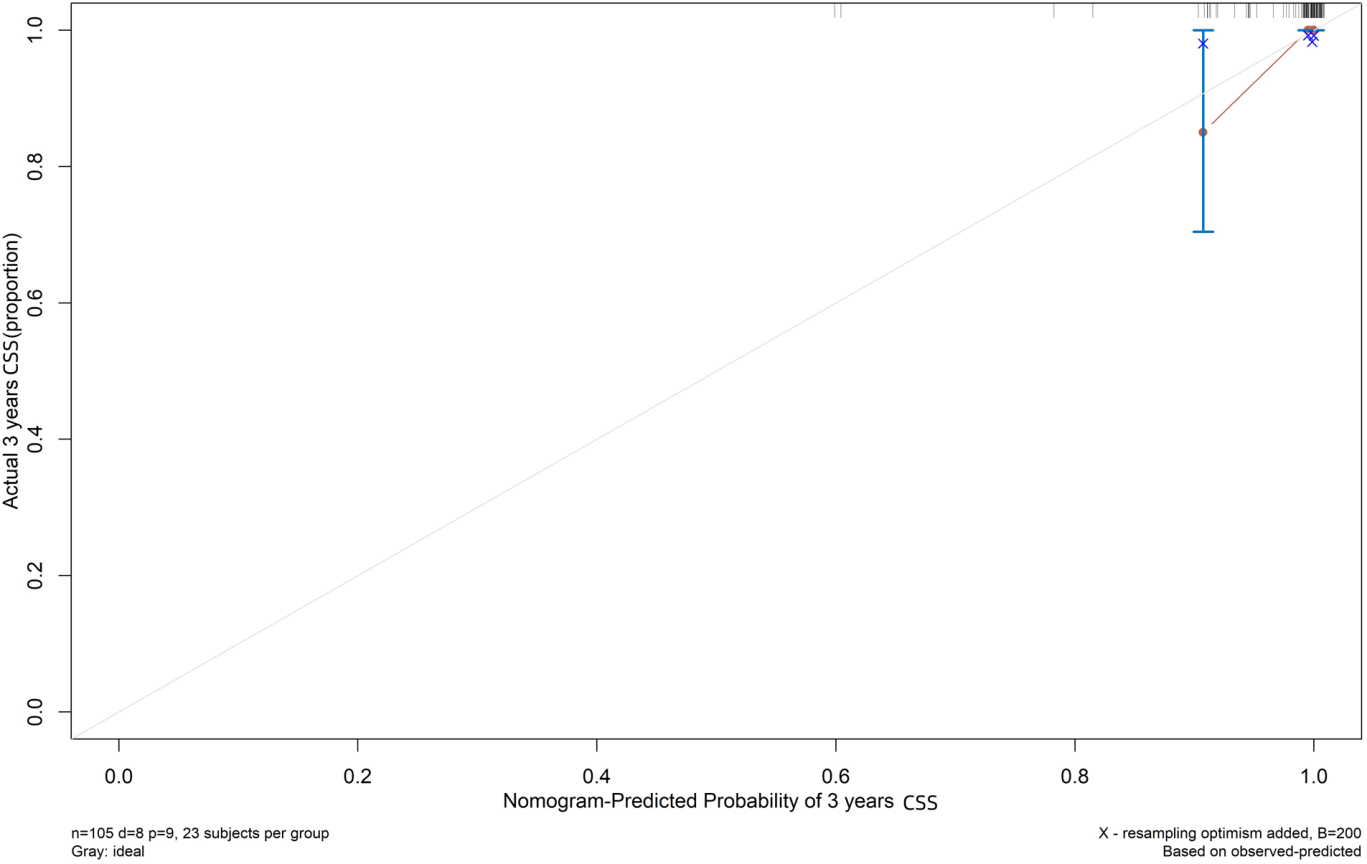
Calibration plot for cause-specific mortality nomogram in TETs. The x-axis and y-axis respectively correspond to the predicted odds of cause-specific survival and the actual observed incidence of cause-specific survival (3-year) of validation group.

Both the training and validation sets were validated for the model. Divided into two groups according to nomorisk: high risk and low risk, then the Kaplan-Meier curves were established and showed a significant difference(Figure 4). In the time-dependent receiver operating characteristic (ROC) curve of the validation group (Figure 5), the areas under the ROC curves (AUCs) at 3-year, 5-year, and 10-year were 0.946, 0.949, and 0.937, respectively, indicating that the prediction accuracy of this nomogram was high at these three time points. The calibration curve (Figure 6) also showed a good predictive ability of the model.

## Discussion

As a relatively rare anterior mediastinal tumor, the incidence of TETs is higher in China (4.09/1 million) than in other areas(1.3∼3.2/1 million) (19). Currently, surgical resection is the best treatment for TETs, and most cases can be cured by surgery (20). At the same time, postoperative radiotherapy and chemotherapy are also widely used. To date, no randomized controlled trials have been conducted to evaluate the effect of postoperative chemotherapy on TETs (15), and the effect of postoperative chemotherapy on patient survival is still controversial. However, for R2 resection and metastatic TETs, postoperative chemotherapy is recommended (21–23). Some studies have proposed prognostic models using the SEER database, but the data in the SEER database are different and missing the surgical methods and postoperative treatment data. Masaoka-Koga staging is the most commonly used method for TETs, and TNM staging was introduced later. As demonstrated by Meurgey A et al. (24), when switching from the Masaoka-Koga stage to TNM stage (AJCC 8th Edition), histological types were associated with tumor stage (3, 25, 26), and the good and significant correlation between them contributes to the prognostic value of WHO classification. Therefore, it is necessary to establish a prognostic model of TETs on TNM stage.

In the establishment of this prediction model, five factors were included: age, histological type, tumor size, myasthenia gravis, and TNM stage. Among them, myasthenia gravis and TNM stage were the variables included for the first time. Age has been reported to be a prognostic factor for TETs (9, 27); however, Yanagiya et al. demonstrated that age and histological type were not meaningful prognostic factors for thymoma compared to stage (28). In this study, the risk of patients ≥50 years of age was significantly higher than that of patients <50 years of age, which suggested that age is a meaningful prognostic factor for TETs, and older patients may have higher possibilities of experiencing worse CSS outcomes. Among the histological types, the risk of B3/Ca was the highest (HR = 14.4, *P* < 0.05), followed by B2 (HR = 8.07, *P* = 0.066), and the results were consistent with the clinical consensus on the prognosis of the pathological subtype. In comparison, the patients with type A/AB/B1 have fewer malignant tumors and longer survival. Tumor size has been shown to be an independent risk factor for prognosis, with larger tumors having higher recurrence and mortality (29), and the patients with tumor size ≥6 cm in this study had a higher risk for mortality (*p* < 0.01). A larger tumor size usually means more difficulties for resection and higher recurrence rates. However, in A/AB/B1 TETs, tumors tend to grow within the membrane, and large tumor diameters may have early TNM stages. In this study, myasthenia gravis was also listed as a risk factor after screening (*p* < 0.05). TETs are often associated with myasthenia gravis (30–32); Tian W et al. (33) believed that patients with myasthenia gravis had smaller tumors and a higher proportion of advanced tumors; and myasthenia gravis was significantly associated with poorer OS and recurrence free survival in TETs. Of course, some studies have concluded that myasthenia gravis affecting neurologic related survival (34).This study indicated that myasthenia gravis is associated with poor prognosis for TET patients, and although its risk in the model is lower than that of other factors, we believe that patients with myasthenia gravis need more attention in postoperative therapy. The surgical margin is an important factor for the prognosis of TET patients and is a measure of the effectiveness of surgical excision. R1/R2 patients tend to be more prone to recurrence and higher mortality.Stages I and II have very high rates of R0 resection, but stages III and IV have much lower rates (50%) and 25%, respectively (5). The stage III prognosis significantly improves after a radical resection, almost reaching a stage I prognosis (35, 36). In the Cox multivariate regression, surgical margin status was not significant (*P* > 0.05). We recognize that it is related to a small amount of R1/R2 data (21,0.06), because of the goal of expanded thymectomy is R0 resection, so we cannot assert that margin status is not a prognostic factor in our study. In Surgical treatment, R0 resection still improves prognosis significantly, especially in advanced patients. TNM stage showed an important prognostic role in the Cox multivariate regression. The risk of stage II was significantly higher than that of stage I, which was not significant in the Masaoka-Koga stage in previous studies. Chiappetta et al. (37) believed that there was no difference in survival between patients with Masaoka-Koga staging in stage I and stage II, while there was a difference in survival between patients with stage I and II after TNM staging. TNM stage and Masaoka-Koga stage have their own advantages and disadvantages in diagnosis and treatment. Masaoka staging concentrates more on the concept of continuous invasion (stage III) and discontinuous progression (stage IV). In contrast, the TNM system respects the localization of the involved area and prioritizes the surgical outcome (38). By the classification of TNM stage, more early stage patients with better prognosis were enrolled in stage I, and the risk of stage II (HR = 14.1, *P* = 0.001) or III/IV (HR = 43.7, *P* < 0.001) was significantly higher. We believed that after surgery, patients with TNM stage could have better performance in the prediction of prognosis, and the nomogram was established based on TNM stage. Our study also analyzed postoperative radiotherapy and postoperative chemotherapy in the multivariate Cox regression model. As a result, postoperative radiotherapy was observed to be a protective factor (*P* = 0.121 > 0.05) but was not statistically significant. We considered postoperative chemotherapy to have marginal statistical significance (*P* = 0.072 > 0.05) because the sample size of the patients (55,0.157) who received postoperative chemotherapy in this study was small. At present, there is still some controversy about the effect of postoperative chemotherapy on the treatment of TETs. In Zhao M's study (15), postoperative chemotherapy was a risk factor in the prediction model. Our analysis yielded the same result, HR = 4.81. Advanced stage, the small sample size, and some patients receiving no standardized chemotherapy cycles may be the reason for this conclusion. Furthermore, lymph node dissection and positive lymph nodes were not considered to be significant prognostic factors in the analysis. Due to the low number of patients with lymph node metastasis within the TET patients, lymph node dissection is still the current surgical controversy (39). Wang et al. (40) reported that the prognosis of patients who did not receive lymph node dissection was significantly worse than that of patients who received lymph node dissection and were positive for lymph node metastasis; however, there was no significant difference in the patients with negative lymph node metastasis. There were few lymph node dissection patients in this study, and the results need to be further confirmed.

Our nomogram is innovative and rational in the following aspects. First, our nomogram is the first method to predict the prognosis of TETs based on TNM stage, which makes the individualized prediction of CSS and individualized treatment guidance possible. Second, many characteristics are involved in our analysis, not only the TNM stage but also other variables such as age, histological type, tumor size, and myasthenia gravis, in patients with TETs. In particular, myasthenia gravis was associated with poor prognosis in the nomogram, which has important clinical significance. Third, as a result of the data from Qilu Hospital and because of the rigorous algorithm, the performance of the nomograms was reliable. In conclusion, our prognostic model is innovative and rational enough to be effective in clinical practice.

However, there are still some limitations of this study. First, compared to the SEER database-based analysis, our analysis has a relatively small sample size, which needs to be extended in the follow-up. Second, as a retrospective study, the nomogram needs to be validated in the next prospective cohort before it can be formally applied in clinical practice. In addition, some factors, such as margin status and postoperative chemotherapy, were not included in the nomogram because of the small sample size, and these factors may also be associated with the prognosis of TETs. Therefore, a more complete model that includes margin status and postoperative treatment is needed in the future. Besides,the surgical approach may also be an important prognostic factor that needs to be explored in subsequent studies. Finally, although the AUCs of the 3-year, 5-year, and 10-year tdROC curves are all greater than 0.9, indicating that the model for CSS has high precision, it is not perfect because approximately 20% of predictions are still wrong. In fact, it is impossible for any predictive model to achieve 100% accuracy, but we will do our best to improve the quality and quantity of data and the reliability of our algorithms to achieve this goal.

## Conclusion

In this study, our prognostic model demonstrated that demographic characteristics, clinical characteristics, and TNM stage were all significantly associated with survival outcomes in TET patients following extended thymectomy. More importantly, we built an accurate and visible nomogram to predict individual CSS in postoperative patients with TETs. The nomogram will help clinicians assess the risk of patients with TETs and guide more individualized treatment.

## Data Availability

The original contributions presented in the study are included in the article/**Supplementary Material**, further inquiries can be directed to the corresponding author/s.
