# Supplementary material for: Nomogram based on TNM stage to predict the prognosis of thymic epithelial tumors (TETs) patients undergoing extended thymectomy
Source: Front Surg. 2023 Mar 3;10:1136166. doi: 10.3389/fsurg.2023.1136166 (PMC10020510; doi:10.3389/fsurg.2023.1136166)
Supplement: Supplementary file 1 [file Datasheet1.docx]

Supplementary Material

# Supplementary Figures and Tables

## Supplementary Figures

**Supplementary Figure 1.** Calibration plot for cause-specific mortality nomogram in Ts and TC. The x-axis and y-axis respectively correspond to the predicted odds of cause-specific survival and the actual observed incidence of cause-specific survival(5-year) of validation group.


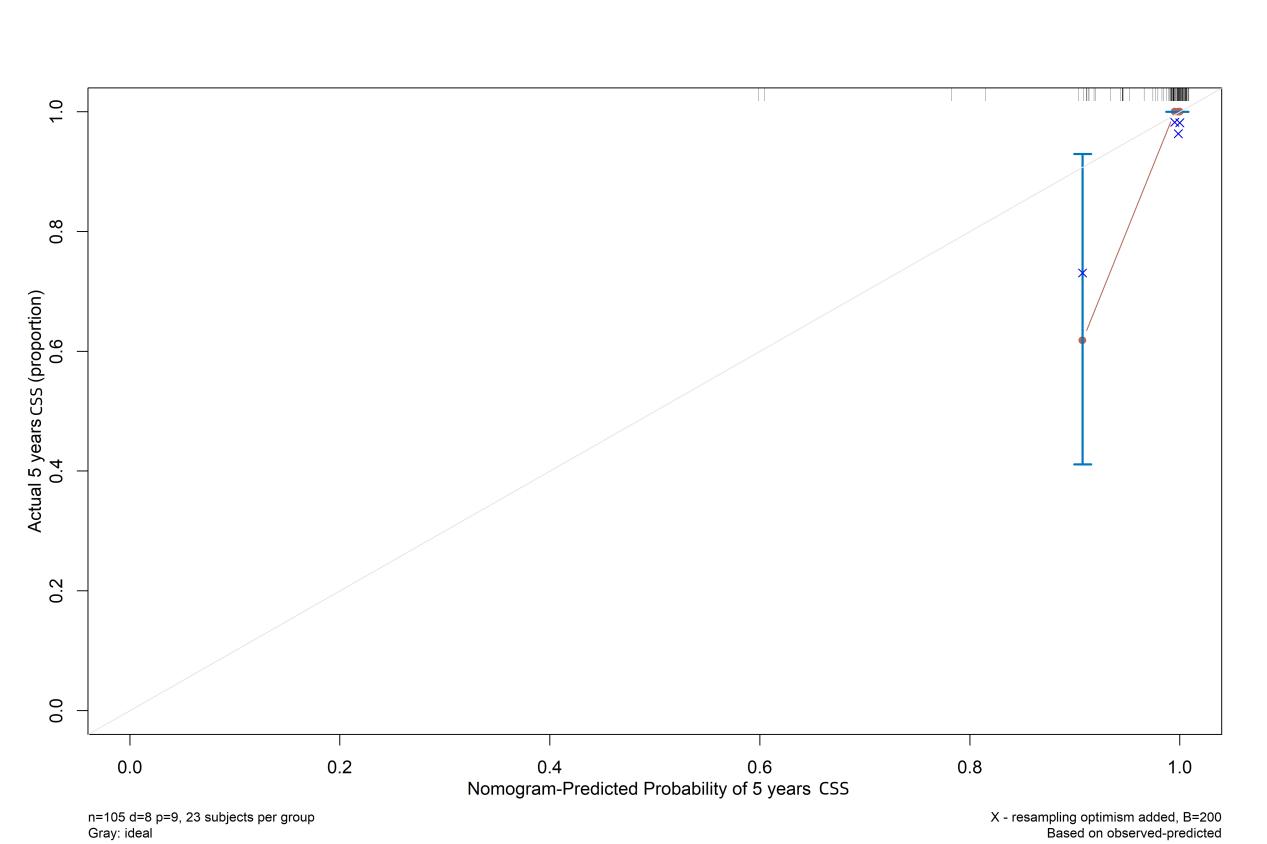


**Supplementary Figure 2.** Calibration plot for cause-specific mortality nomogram in Ts and TC. The x-axis and y-axis respectively correspond to the predicted odds of cause-specific survival and the actual observed incidence of cause-specific survival(8-year) of validation group.


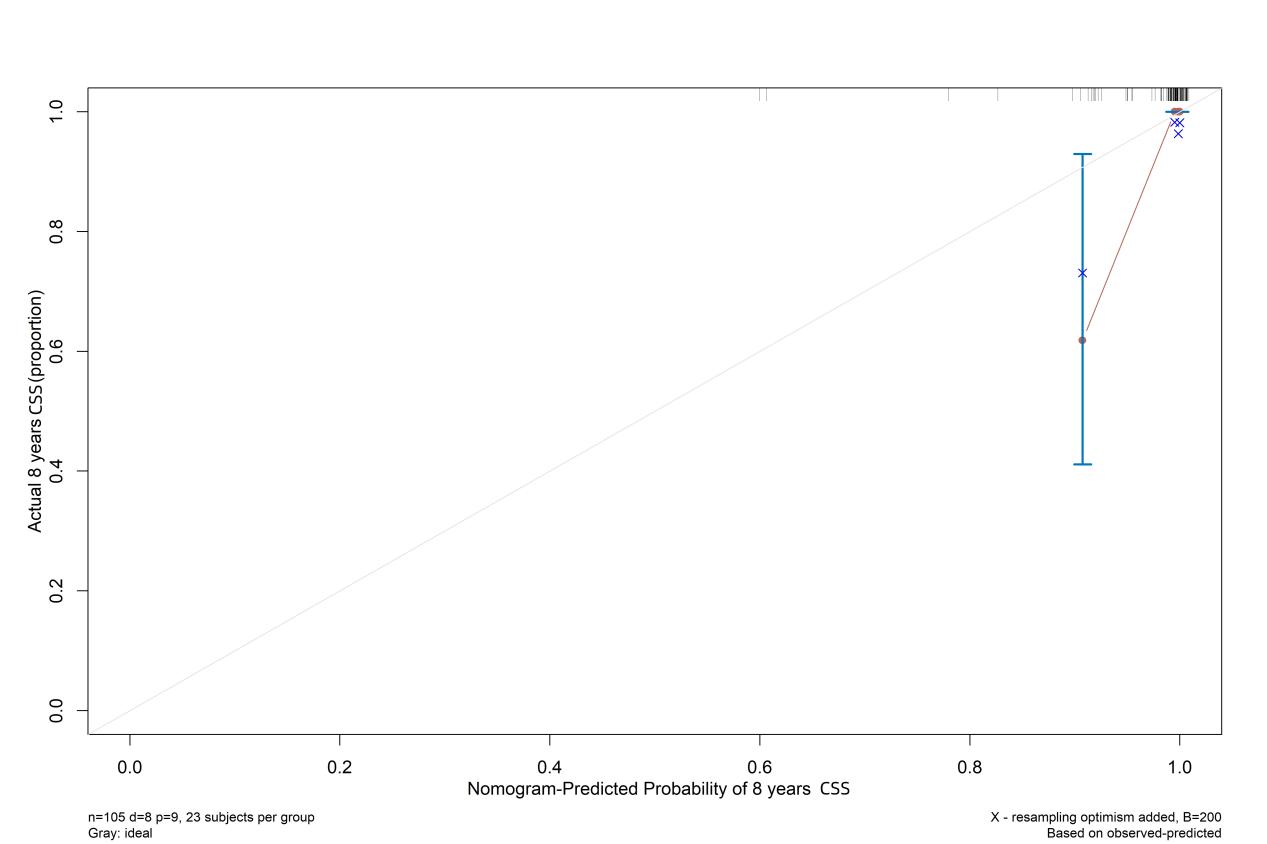


## Supplementary Tables

**Supplementary Table 1.** Baseline Characteristics of Inclusion Group and Exclusion Group.

| Variable | Level | class = 1 (n=350) | class = 2 (n=28) | P Value |
| --- | --- | --- | --- | --- |
| Age | <50 | 140(40.0) | 11(39.3) | 1.00 |
|  | ≥50 | 210 (60.0) | 17 (60.7) |  |
| Sex | Male | 177(50.6) | 15(53.6) | 0.77 |
|  | Female | 173(49.4) | 13 (46.4) |  |
| Path | A/AB/B1 | 141(40.3) | 12(42.9) | 0.88 |
|  | B2 | 106 (30.3) | 9(32.1) |  |
|  | B3/CA | 103 (29.4) | 7(25.0) |  |
| Size | <6 | 221 (63.1) | 16 (57.1) | 0.67 |
|  | ≥6 | 129(36.9) | 12(42.9) |  |
| Hydrothorax | No | 273 (78.0) | 21 (75.0) | 0.90 |
|  | Yes | 77 (22.00) | 7 (25.0) |  |
| Lymph_node_dissection | No | 292 (83.4) | 25 (89.3) | 0.42 |
|  | Yes | 58(16.6) | 3 (10.7) |  |
| Positive_lymph_node | No | 345 (98.6) | 28(100.0) | 0.52 |
|  | Yes | 5(1.4) | 0(0.0) |  |
| Myasthenia | No | 222 (63.4) | 18 (64.3) | 1.00 |
|  | Yes | 128 (36.6) | 10 (35.7) |  |
| Margin | R0 | 329（94.0) | 26(92.9) | 0.81 |
|  | R1/R2 | 21 (6.0) | 2 (7.1) |  |
| Masaoka | I/IIa | 202 (55.7) | 20 (71.4) | 0.14 |
|  | IIb | 50 (14.3) | 5 (17.9) |  |
|  | III/IV | 98 (28.0) | 3(10.7) |  |
| TNM_stage | I | 269 (76.9) | 22 (78.6) | 0.67 |
|  | II | 55 (15.7) | 3(10.7) |  |
|  | III/IV | 26 (9.4) | 3(10.7) |  |

TNM, Tumor Node Metastasis;Continuous variables are presented as median [IQR]. Categorical variables are presented as n (%).

**Supplementary Table 2.** Baseline Characteristics of Training Group and Validation Group.

| Variable | Level | class = 1 (n=245) | class = 2 (n=105) | Total (n=350) |
| --- | --- | --- | --- | --- |
| Age | <50 | 100 (40.8) | 40 (38.10) | 140 (40.0) |
|  | ≥0 | 145 (59.2) | 65 (61.90) | 210 (60.0) |
| Sex | Male | 119 (48.6) | 58 (55.24) | 177 (50.6) |
|  | Female | 126 (51.4) | 47 (44.76) | 173 (49.4) |
| Path | A/AB/B1 | 92 (37.6) | 49 (46.67) | 141 (40.3) |
|  | B2 | 79 (32.2) | 27 (25.71) | 106 (30.3) |
|  | B3/CA | 74 (30.2) | 29 (27.62) | 103 (29.4) |
| Size | <6 | 158 (64.5) | 63 (60.00) | 221 (63.1) |
|  | ≥6 | 87 (35.5) | 42 (40.00) | 129 (36.9) |
| Hydrothorax | No | 190 (77.6) | 83 (79.05) | 273 (78.0) |
|  | Yes | 55 (22.4) | 22 (20.95) | 77 (22.0) |
| Lymph_node_dissection | No | 201 (82.0) | 91 (86.67) | 292 (83.4) |
|  | Yes | 44 (18.0) | 14 (13.33) | 58 (16.6) |
| Positive_lymph_node | No | 241 (98.4) | 104 (99.05) | 345 (98.6) |
|  | Yes | 4 (1.6) | 1 (0.95) | 5 (1.4) |
| Myasthenia | No | 148 (60.4) | 74 (70.48) | 222 (63.4) |
|  | Yes | 97 (39.6) | 31 (29.52) | 128 (36.6) |
| Margin | R0 | 229 (93.5) | 100 (95.24) | 329 (94.0) |
|  | R1/R2 | 16 (6.5) | 5 (4.76) | 21 (6.0) |
| Masaoka | I/IIa | 144 (58.8) | 58 (55.24) | 202 (57.7) |
|  | IIb | 30 (12.2) | 20 (19.05) | 50 (14.3) |
|  | III/IV | 71 (29.0) | 27 (25.71) | 98 (28.0) |
| TNM_stage | I | 184 (75.1) | 85 (80.95) | 269 (76.9) |
|  | II | 39 (15.9) | 16 (15.24) | 55 (15.7) |
|  | III/IV | 22 (9.0) | 4 (3.81) | 26 (7.4) |

TNM, Tumor Node Metastasis;Continuous variables are presented as median [IQR]. Categorical variables are presented as n (%).
